# Hydatid of Liver

**Published:** 1915-03

**Authors:** 


**Hydatid of Liver.** J. P. S. Jamison of Eketahuna, New Zealand Med. Jour., Dec., 1914, reports a sheep-farmer of 69, with herpes over the liver, apparently due to irritation of a cyst, apparently involving the left lobe of the liver and at first diagnosed as gall stones from jaundice and acholic stools. Marked retching and hiccough produced rupture into the stomach and vomiting of 2-3 pints of appearently purulent matter containing hydatid membrane and "strongly odorous of b. coli." This spontaneous evacuation occurred just before appointment for operation and resulted in apparent cure.

